# Supplementary material for: Two Important Anticancer Mechanisms of Natural and Synthetic Chalcones
Source: Int J Mol Sci. 2022 Sep 30;23(19):11595. doi: 10.3390/ijms231911595 (PMC9570335; doi:10.3390/ijms231911595)
Supplement: Supplementary file 1 [file ijms-23-11595-s001.zip › Figures S1 and S2.pdf]

# Supplementary Figure

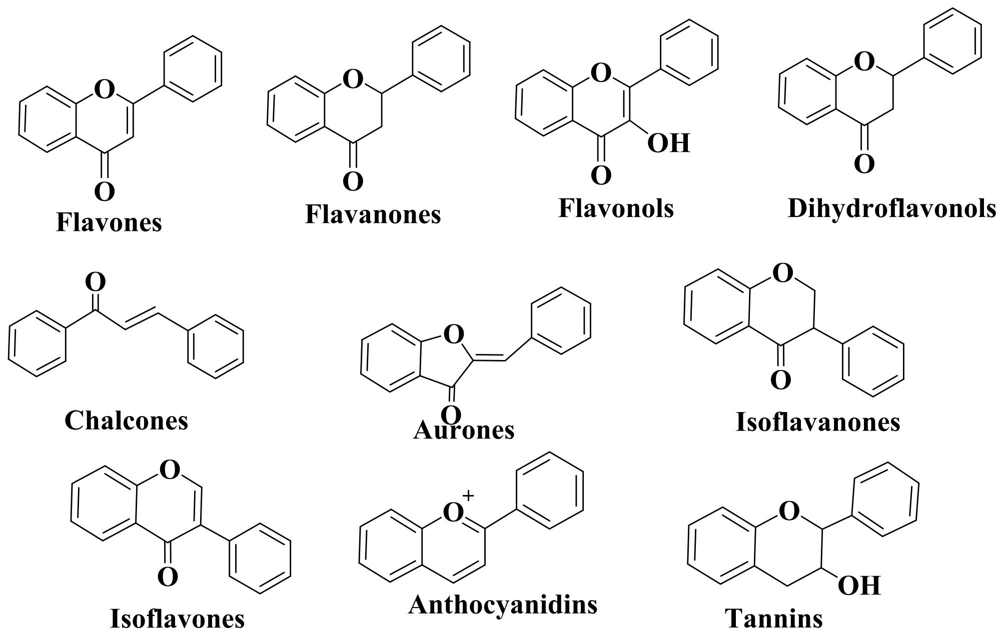


**Figure S1.** Basic structure of flavonoids.

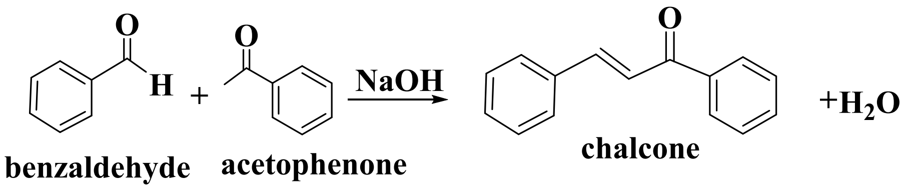


**Figure S2.** Claisen-Schmidt reaction.
