# Supplementary material for: Two Important Anticancer Mechanisms of Natural and Synthetic Chalcones
Source: Int J Mol Sci. 2022 Sep 30;23(19):11595. doi: 10.3390/ijms231911595 (PMC9570335; doi:10.3390/ijms231911595)
Supplement: Supplementary file 1 [file ijms-23-11595-s001.zip › Table S1.pdf]

**Table S1.** Chemical structures of flavonoids		
	Structure	Name
1	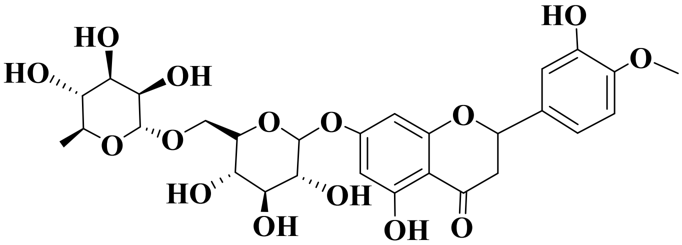	Hesperidin
2	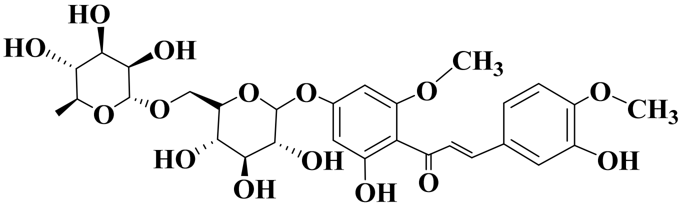	Hesperidin methyl chalcone
3	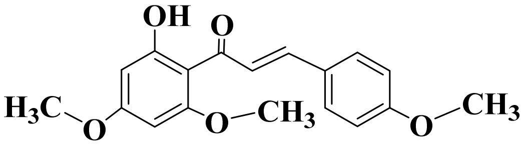	Flavokawain A
4	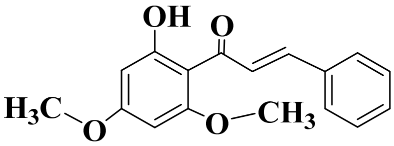	Flavokawain B
5	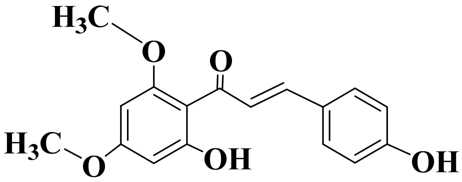	Flavokawain C
6	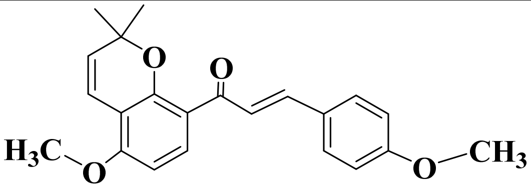	Millepachine
7	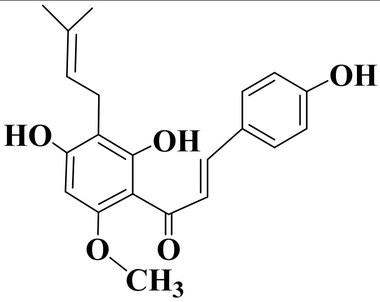	Xanthohumol

8	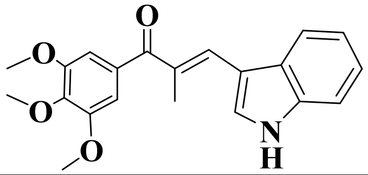	
9	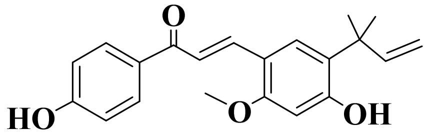	Licochalcone A
10	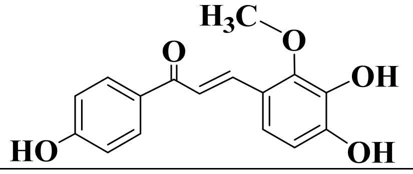	Licochalcone B
11	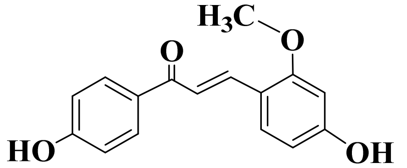	Echinatin
12	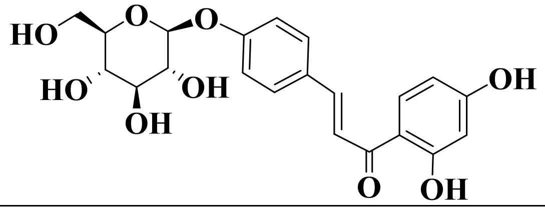	Isoliquiritin
13	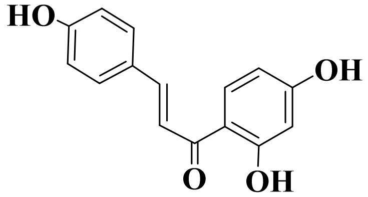	Isoliquiritigenin
14-57	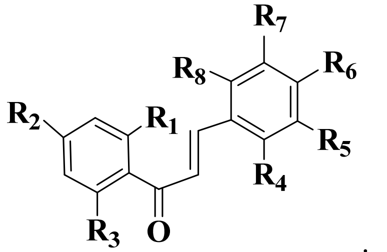 .	14.R_1, R_3=OMe
		15.R_1, R_3, R_4=OMe
		16.R_1, R_3, R_5=OMe
		17. R_1, R_3, R_8=OMe
		18. R_1, R_3, R_4, R_6, R_8=OMe
		19. R_1, R_3, R_6, R_7, R_8=OMe
		20. R_1, R_3,R_5, R_6, R_8=OMe
		21. R_1, R_3,R_5, R_6, R_7=OMe
		22. R_1, R_3=OEt, R_4, R_6, R_8=OMe
		23. R_1, R_3=OEt, R_4, R_8=OMe
		24. R_1, R_3=OEt, R_4, R_6=OMe
		25. R_1, R_2, R_6, R_8=OMe
		26. R_1, R_2, R_3,R_4, R_6, R_8=OMe
		27. R_1, R_2, R_3,R_4, R_6 =OMe
		28. R_1, R_2, R_3,R_4, R_8 =OMe
		29. R_1, R_2, R_3,R_4, R_8 =OEt

		30. R_4, R_8 =OMe
		31. R_1=OH, R_2, R_5, R_7 =OMe
		32. R_1=OH, R_2, R_6, R_7,R_8 =OMe
		33. R_1=OH, R_2, R_6, R_7 =OMe
		34. R_1=OH, R_2, R_4,R_6, R_8 =OMe
		35. R_1=OH, R_2, R_7, R_8 =OMe
		36. R_1=OH, R_2, R_5, R_8 =OMe
		37. R_1=OH, R_2, R_5,R_6, R_7 =OMe
		38. R_1=OH, R_2, R_4, R_8 =OMe
		39. R_1=OH, R_2, R_6, R_8 =OMe
		40.R_3=OH, R_1, R_2, R_4, R_8=OMe
		41. R_3=OH, R_1, R_2, R_5, R_7=OMe
		42. R_3=OH, R_1, R_2 =OMe
		43. R_3=OH, R_1, R_2, R_4 =OMe
		44. R_3=OH, R_1, R_2, R_5=OMe
		45. R_3=OH, R_1, R_2, R_4, R_6 =OMe
		46. R_3=OH, R_1, R_2, R_5, R_6, R_7 =OMe
		47. R_3=OH, R_1, R_2, R_6, R_7, R_8 =OMe
	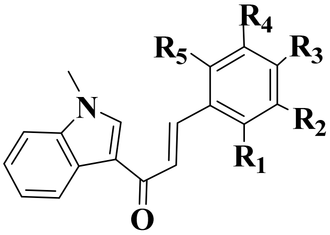	48.R_1,R_3=OMe
		49.R_2,R_3,R_4=OMe
		50.R_1,R_3,R_5=OMe
		51. R_1,R_5=OMe
	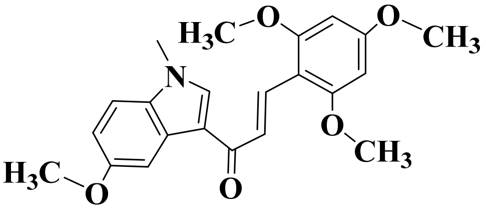	52
	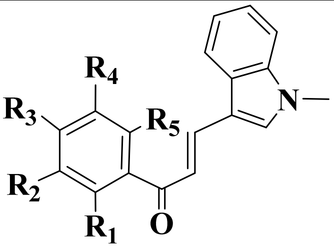	53.R_1,R_3=OMe
		54.R_3=OMe
		55.R_1,R_3,R_5=OMe
		56. R_1, R_5=OMe
	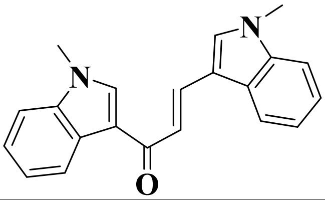	57.

58-61	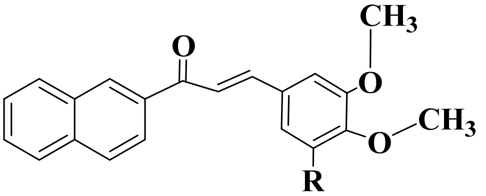	58.R=Cl
		59.R=Br
		60.R=I
		61.R=OMe
62-83	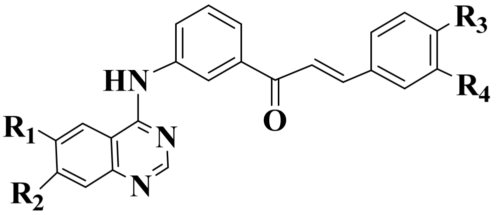	62.R_1,R_2=H; R_3,R_4=OMe
		63.R_1, R_2, R_4=H; R_3=OMe
		64.R_1,R_2,R_3,R_4=OMe
		65. R_1,R_2,R_3 =OMe; R_4=H
	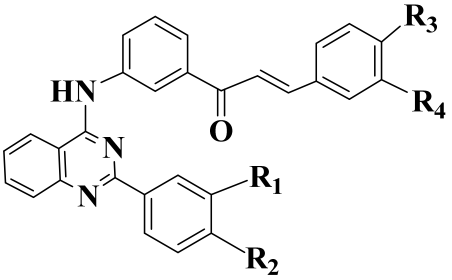	66. R_1,R_2=H; R_3,R_4=OMe
		67. R_1, R_2, R_4=H; R_3=OMe
		68. R_1, R_2, R_3=H; R_4=OMe
		69. R_1,R_2,R_3,R_4=OMe
		70. R_1,R_2,R_3 =OMe; R_4=H
		71.R_1,R_2,R_4=OMe; R_3=H
	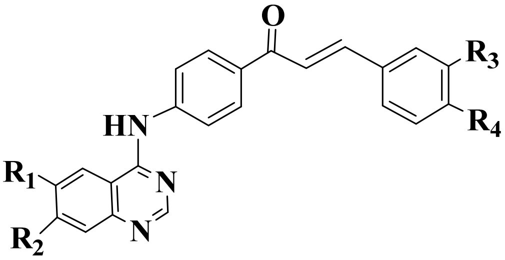	72. R_1,R_2=H; R_3,R_4=OMe
		73. R_1, R_2, R_4=H; R_3=OMe
		74. R_1,R_2,R_3,R_4=OMe
		75. R_1,R_2,R_3 =OMe; R_4=H
	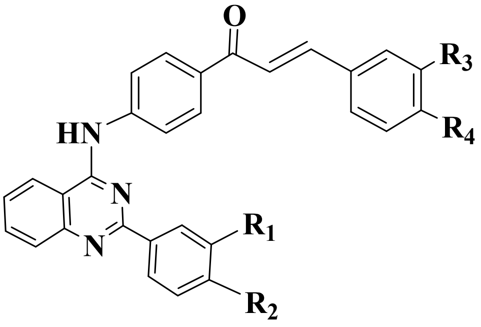	76. R_1,R_2=H; R_3,R_4=OMe
		77. R_1, R_2, R_4=H; R_3=OMe
		78. R_1, R_2, R_3=H; R_4=OMe
		79. R_1,R_2,R_3,R_4=H
		80. R_1,R_2,R_3,R_4=OMe
		81. R_1,R_2,R_3 =OMe; R_4=H
		82. R_1,R_2, R_4=OMe; R_3=H
		83. R_1,R_2 =OMe; R_3,R_4=H
84-95	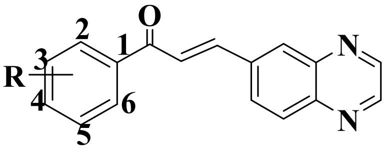	84.R=4-OMe
		85.R=2,5-diOMe
		86.R=3,4-diOMe
		87.R=2,4-diOMe
		88.R=3,4,5-triOMe
		89.R=3-OMe, 4-OH
		90.R=2,4,5-triOMe
		91.R=3,5-diOMe
		92.R=2-OMe

		93.R=3,5-diOMe, 4-OH
		94.R=3-OMe
		95.R=2,3,4-triOMe
96-131	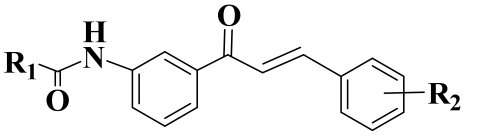	96.R_1=Ph, R_2=3,4-diOMe
		97.R_1=4-$O_2NC_6H_4$, R_2=3,4-diOMe
		98.R_1=3-BrC_6H_4, R_2=3,4-diOMe
	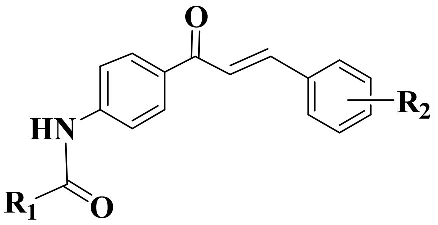	99. R_1=Ph, R_2=3,4-diOMe
		100. R_1=2-$O_2NC_6H_4$, R_2=3,4-diOMe
		101. R_1=3-$O_2NC_6H_4$, R_2=3,4-diOMe
		102. R_1=4-$O_2NC_6H_4$, R_2=3,4-diOMe
		103. R_1=3-BrC_6H_4, R_2=3,4-diOMe
		104. R_1=4-BrC_6H_4, R_2=3,4-diOMe
		105. R_1=3-ClC_6H_4, R_2=3,4-diOMe
		106. R_1=4-ClC_6H_4, R_2=3,4-diOMe
		107. R_1=3,4-diClC_6H_4, R_2=3,4-diOMe
		108. R_1=2-FC_6H_4, R_2=3,4-diOMe
		109. R_1=4-NCC_6H_4, R_2=3,4-diOMe
		110. R_1=4-$F_3CC_6H_4$, R_2=3,4-diOMe
		111. R_1=2-$MeOC_6H_4$, R_2=3,4-diOMe
		112. R_1=3-$MeOC_6H_4$, R_2=3,4-diOMe
		112. R_1=4-$MeOC_6H_4$, R_2=3,4-diOMe
		114. R_1=3,4-di$MeOC_6H_3$, R_2=3,4-diOMe
		115. R_1=3,4,5-tri$MeOC_6H_2$, R_2=3,4-diOMe
	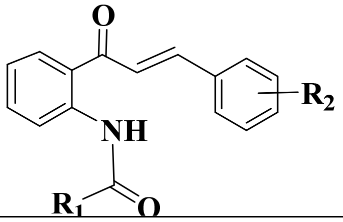	116. R_1=Ph, R_2=3,4-diOMe
		117. R_1=3-ClC_6H_4, R_2=3,4-diOMe
		118. R_1=4-ClC_6H_4, R_2=3,4-diOMe
		119. R_1=2-$O_2NC_6H_4$, R_2=3,4-

		diOMe
		120. R_1=3-$O_2NC_6H_4$, R_2=3,4-diOMe
		121. R_1=2-$MeOC_6H_4$, R_2=3,4-diOMe
		122. R_1=3-$MeOC_6H_4$, R_2=3,4-diOMe
		123. R_1=3-quinalinyl, R_2=3,4-diOMe
		124. R_1=1-naphtyl, R_2=3,4-diOMe
		125. R_1=3-pyridil, R_2=3,4-diOMe
		126. R_1=7-methoxy-2-oxo-3-chromenyl, R_2=3,4-diOMe
		127. R_1=2-thienyl, R_2=3,4-diOMe
		128. R_1=3-ClC_6H_4, R_2=3-Cl
		129. R_1=3,4-di$MeOC_6H_3$, R_2=3-Cl
		130. R_1=2-thienyl, R_2=3-OMe, 4-F
		131. R_1=3-ClC_6H_4, R_2=3-OMe, 4-F
132 - 137	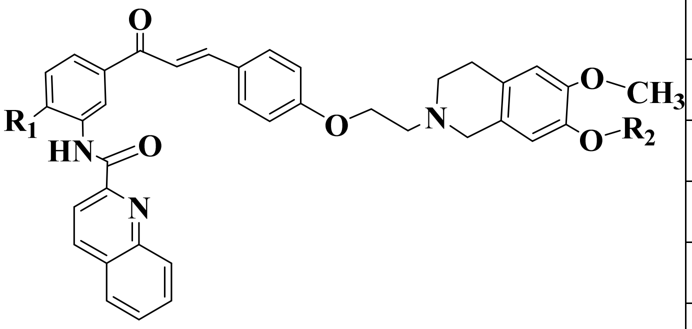	132. R_1=H, R_2=OMe
		133. R_1=H, R_2=-CH_2-CH_2-OCH_3;
		134. R_1=H, R_2=$(CH_2\text{-}CH_2\text{-}O)_3CH_3$
		135. R_1=Me, R_2=OMe;
		136. R_1=Me, R_2=-CH_2-CH_2-OCH_3
		137. R_1=Me, R_2: $(CH_2\text{-}CH_2\text{-}O)_3CH_3$
138	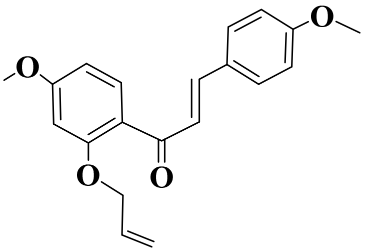	138
139	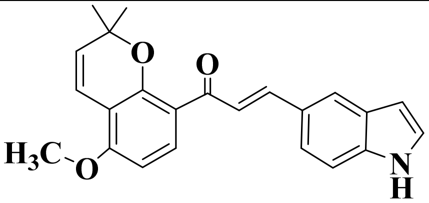	

140	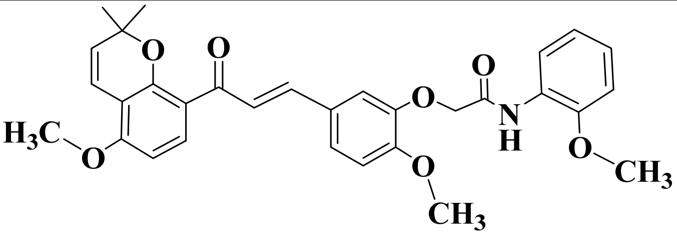	
141	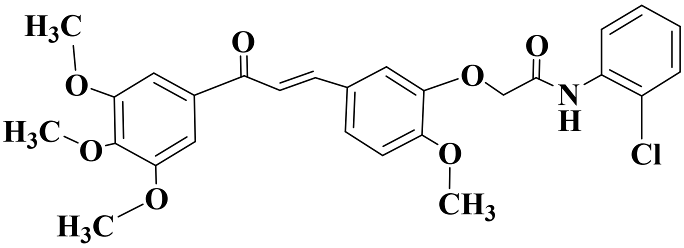	
142<br>-<br>161	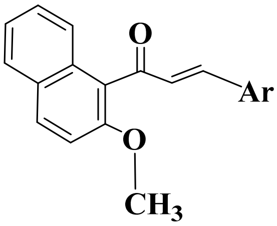	142.Ar=3-hydroxy-4-methoxyphenyl
		143.Ar=4-bromophenyl
		144.Ar=2,3,4-trimethoxyphenyl
		145.Ar=2-bromophenyl
		146.Ar=naphthalen-1-yl
		147. Ar=naphthalen-2-yl
		148.Ar=3-bromo-4-methoxyphenyl
		149.Ar=3-methoxyphenyl
		150.Ar=4-methoxy-3-nitrophenyl
		151.Ar=thiophen-2-yl
		152.Ar=4-chlorophenyl
		153.Ar=2,4,5-trimethoxyphenyl
		154.Ar=2-fluorophenyl
		155.Ar=phenyl
		156.Ar=3-fluorophenyl
		157.Ar=3,4,5-trimethoxyphenyl
		158.Ar=2-methoxyphenyl
		159.Ar=4-(dimethylamino)phenyl
		160.Ar=4-(diethylamino)phenyl
		161.Ar=3-amino-4-methoxyphenyl
162<br>-<br>177	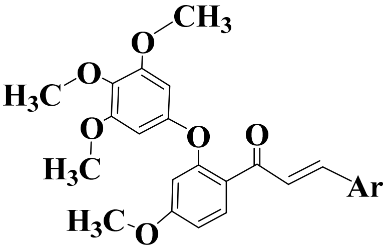	162.Ar=4-chlorophenyl
		163.Ar=4-methoxyphenyl
		164.Ar=4-(diethylamino)phenyl
		165. Ar=4-(dimethylamino)phenyl
		166. Ar=3,4,5-trimethoxyphenyl
		167.Ar=phenyl

		168.Ar=2-fluorophenyl
		169.Ar=naphtalen-1-yl
		170.Ar=4-bromophenyl
		171. Ar=4-fluorophenyl
		172. Ar=3-fluorophenyl
		173. Ar=2,3,4-trimethoxyphenyl
		174.Ar=4-methoxy-3-nitrophenyl
		175.Ar= naphtalen-2-yl
		176.Ar=3-chlorophenyl
		177.Ar=3-amino-4-methoxyphenyl
178	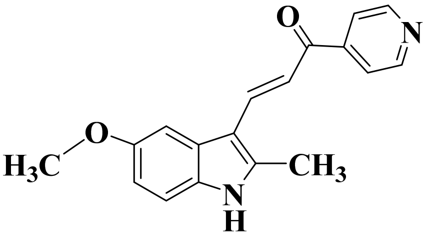	178
179<br>-<br>187	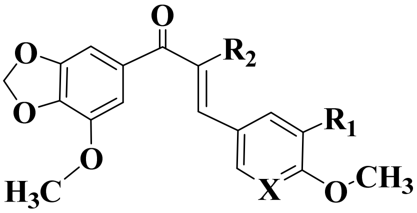	179.R_1=OH, R_2=H, X=C
		180.R_1=F, R_2=H, X=C
		181.R_1=H, R_2=H, X=N
		182.R_1=NO_2, R_2=H, X=C
		183.R_1=NH_2, R_2=H, X=C
		184.R_1=OH, R_2=CH_3, X=C
		185.R_1=NH_2, R_2=CH_3, X=C
		186.R_1=NH-Ser, R_2=CH_3, X=C
		187.R_1=NH-Pro-Lys, R_2=CH_3, X=C
188	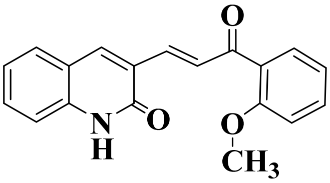	
189	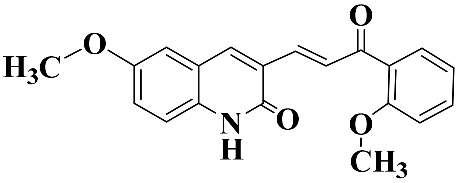	
190<br>-<br>214	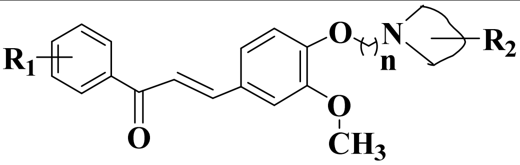	190.R_1=4-OMe, n=3, R_2=piperidinyl
		191. R_1=2-OH, n=3, R_2=piperidinyl
		192. R_1=4-Br, n=3, R_2=piperidinyl
		193. R_1=H, n=4, R_2=piperidinyl

		194. R_1=4-Br, n=4, R_2=piperidinyl
		195. R_1=4-Cl, n=4, R_2=piperidinyl
		196. R_1=2,4-diCl, n=4, R_2=piperidinyl
		197. R_1=4-OMe, n=2, R_2=morpholinyl
		198. R_1=2-Br, n=2, R_2=morpholinyl
		199. R_1=2,4-diCl, n=2, R_2=morpholinyl
		200. R_1=4-CF_3, n=2, R_2=morpholinyl
		201. R_1=4-OMe, n=3, R_2=morpholinyl
		202. R_1=4-OMe, n=3, R_2=2,6-diOMe morpholinyl
		203. R_1=2-OMe, n=3, R_2=2,6-diOMe morpholinyl
		204. R_1=2,4-diCl, n=3, R_2=2,6-diOMe morpholinyl
		205. R_1=4-Me, n=3, R_2=2,6-diOMe morpholinyl
		206. R_1=4-OMe, n=3, R_2=indolinyl
		207. R_1=2,4-diCl, n=3, R_2=indolinyl
		208. R_1=H, n=3, R_2=1,2,3,4-tetrahydroquinolinyl
		209. R_1=4-OMe, n=3, R_2=1,2,3,4-tetrahydroquinolinyl
		210. R_1=4-Br, n=3, R_2=1,2,3,4-tetrahydroquinolinyl
		211. R_1=4-Cl, n=3, R_2=1,2,3,4-tetrahydroquinolinyl
		212. R_1=2,4-diCl, n=3, R_2=1,2,3,4-tetrahydroquinolinyl
		213. R_1=4-NH_2, n=3, R_2=1,2,3,4-tetrahydroquinolinyl
		214. R_1=4-OMe, n=3, R_2=3,5-diMe-1H-pyrazolyl

215 - 232	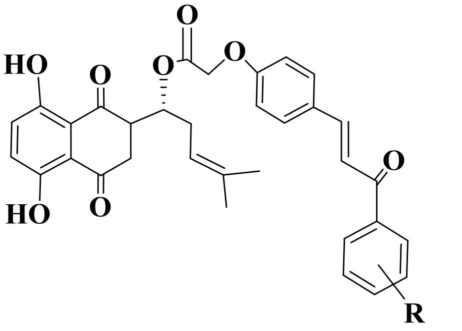	215.R=H
		216.R=4-OMe
		217.R=3,4,5-triOMe
		218.R=4-CF_3
		219.R=2,3,4-triCl
		220.R=2- CF_3
		221.R=2-OMe
		222.R=3- CF_3
		223.R=4-Et
		224.R=2-F
		225.R=3,5-diCF_3
		226.R=3-OCF_3
		227.R=3-OMe
		228.R=2-Me
		229.R=2-Cl
		230.R=2-Br
		231.R=3-CH_3
		232.R=4- CH_3
233	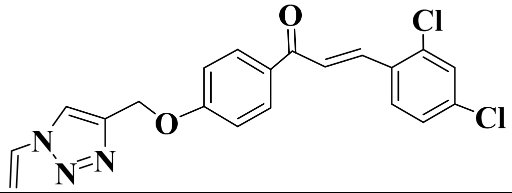	
