# Supplementary material for: Two Important Anticancer Mechanisms of Natural and Synthetic Chalcones
Source: Int J Mol Sci. 2022 Sep 30;23(19):11595. doi: 10.3390/ijms231911595 (PMC9570335; doi:10.3390/ijms231911595)
Supplement: Supplementary file 1 [file ijms-23-11595-s001.zip › Table S2.pdf]

**Table S2.** The name and chemical formula of compounds		
Comp	Name	Chemical Formula
1	5-hydroxy-2-(3-hydroxy-4-methoxyphenyl)-7-(((3R,4S,5S,6S)-3,4,5-trihydroxy-6-((((2R,3R,4R,5R,6S)-3,4,5-trihydroxy-6-methyltetrahydro-2H-pyran-2-yl)oxy)methyl)tetrahydro-2H-pyran-2-yl)oxy)chroman-4-one	$C_{28}H_{34}O_{15}$
2	(E)-3-(3-hydroxy-4-methoxyphenyl)-1-(2-hydroxy-6-methoxy-4-(((3R,4S,5S,6S)-3,4,5-trihydroxy-6-((((2R,3R,4R,5R,6S)-3,4,5-trihydroxy-6-methyltetrahydro-2H-pyran-2-yl)oxy)methyl)tetrahydro-2H-pyran-2-yl)oxy)phenyl)prop-2-en-1-one	$C_{29}H_{36}O_{15}$
3	(E)-1-(2-hydroxy-4,6-dimethoxyphenyl)-3-(4-methoxyphenyl)prop-2-en-1-one	$C_{18}H_{18}O_5$
4	(E)-1-(2-hydroxy-4,6-dimethoxyphenyl)-3-phenylprop-2-en-1-one	$C_{17}H_{16}O_4$
5	(E)-1-(2-hydroxy-4,6-dimethoxyphenyl)-3-(4-hydroxyphenyl)prop-2-en-1-one	$C_{17}H_{16}O_5$
6	(E)-1-(5-methoxy-2,2-dimethyl-2H-chromen-8-yl)-3-(4-methoxyphenyl)prop-2-en-1-one	$C_{22}H_{22}O_4$
7	(E)-1-(2,4-dihydroxy-6-methoxy-3-(3-methylbut-2-en-1-yl)phenyl)-3-(4-hydroxyphenyl)prop-2-en-1-one	$C_{21}H_{22}O_5$
8	(E)-3-(1H-indol-3-yl)-2-methyl-1-(3,4,5-trimethoxyphenyl)prop-2-en-1-one	$C_{21}H_{21}NO_4$
9	(E)-3-(4-hydroxy-2-methoxy-5-(2-methylbut-3-en-2-yl)phenyl)-1-(4-hydroxyphenyl)prop-2-en-1-one	$C_{21}H_{22}O_4$
10	(E)-3-(3,4-dihydroxy-2-methoxyphenyl)-1-(4-hydroxyphenyl)prop-2-en-1-one	$C_{16}H_{14}O_5$
11	(E)-3-(4-hydroxy-2-methoxyphenyl)-1-(4-hydroxyphenyl)prop-2-en-1-one	$C_{16}H_{14}O_4$
12	(E)-1-(2,4-dihydroxyphenyl)-3-(4-(((2S,3R,4S,5S,6R)-3,4,5-trihydroxy-6-(hydroxymethyl)tetrahydro-2H-pyran-2-yl)oxy)phenyl)prop-2-en-1-one	$C_{21}H_{22}O_9$
13	(E)-1-(2,4-dihydroxyphenyl)-3-(4-hydroxyphenyl)prop-2-en-1-one	$C_{15}H_{12}O_4$
14	(E)-1-(2,6-dimethoxyphenyl)-3-phenylprop-2-en-1-one	$C_{17}H_{16}O_3$
15	(E)-1-(2,6-dimethoxyphenyl)-3-(2-methoxyphenyl)prop-2-en-1-one	$C_{18}H_{18}O_4$
16	(E)-1-(2,6-dimethoxyphenyl)-3-(3-methoxyphenyl)prop-2-en-1-one	$C_{18}H_{18}O_4$
17	(E)-1-(2,6-dimethoxyphenyl)-3-(2-methoxyphenyl)prop-2-en-1-one	$C_{18}H_{18}O_4$
18	(E)-1-(2,6-dimethoxyphenyl)-3-(2,4,6-trimethoxyphenyl)prop-2-en-1-one	$C_{20}H_{22}O_6$
19	(E)-1-(2,6-dimethoxyphenyl)-3-(2,3,4-trimethoxyphenyl)prop-2-en-1-one	$C_{20}H_{22}O_6$

20	(E)-1-(2,6-dimethoxyphenyl)-3-(2,4,5-trimethoxyphenyl)prop-2-en-1-one	$C_{20}H_{22}O_6$
21	(E)-1-(2,6-dimethoxyphenyl)-3-(3,4,5-trimethoxyphenyl)prop-2-en-1-one	$C_{20}H_{22}O_6$
22	(E)-1-(2,6-diethoxyphenyl)-3-(2,4-dimethoxyphenyl)prop-2-en-1-one	$C_{21}H_{24}O_5$
23	(E)-1-(2,6-diethoxyphenyl)-3-(2,6-dimethoxyphenyl)prop-2-en-1-one	$C_{21}H_{24}O_5$
24	(E)-1-(2,6-diethoxyphenyl)-3-(2,4-dimethoxyphenyl)prop-2-en-1-one	$C_{21}H_{24}O_5$
25	(E)-1,3-bis(2,4-dimethoxyphenyl)prop-2-en-1-one	$C_{19}H_{20}O_5$
26	(E)-1,3-bis(2,4,6-trimethoxyphenyl)prop-2-en-1-one	$C_{21}H_{24}O_7$
27	(E)-3-(2,4-dimethoxyphenyl)-1-(2,4,6-trimethoxyphenyl)prop-2-en-1-one	$C_{20}H_{22}O_6$
28	(E)-3-(2,6-dimethoxyphenyl)-1-(2,4,6-trimethoxyphenyl)prop-2-en-1-one	$C_{20}H_{22}O_6$
29	(E)-3-(2,6-diethoxyphenyl)-1-(2,4,6-triethoxyphenyl)prop-2-en-1-one	$C_{25}H_{32}O_6$
30	(E)-3-(2,6-dimethoxyphenyl)-1-phenylprop-2-en-1-one	$C_{17}H_{16}O_3$
31	(E)-3-(3,5-dimethoxyphenyl)-1-(2-hydroxy-4-methoxyphenyl)prop-2-en-1-one	$C_{18}H_{18}O_5$
32	(E)-1-(2-hydroxy-4-methoxyphenyl)-3-(2,3,4-trimethoxyphenyl)prop-2-en-1-one	$C_{19}H_{20}O_6$
33	(E)-3-(3,4-dimethoxyphenyl)-1-(2-hydroxy-4-methoxyphenyl)prop-2-en-1-one	$C_{18}H_{18}O_5$
34	(E)-1-(2-hydroxy-4-methoxyphenyl)-3-(2,4,6-trimethoxyphenyl)prop-2-en-1-one	$C_{19}H_{20}O_6$
35	(E)-3-(2,3-dimethoxyphenyl)-1-(2-hydroxy-4-methoxyphenyl)prop-2-en-1-one	$C_{18}H_{18}O_5$
36	(E)-3-(2,5-dimethoxyphenyl)-1-(2-hydroxy-4-methoxyphenyl)prop-2-en-1-one	$C_{18}H_{18}O_5$
37	(E)-1-(2-hydroxy-4-methoxyphenyl)-3-(3,4,5-trimethoxyphenyl)prop-2-en-1-one	$C_{19}H_{20}O_6$
38	(E)-3-(2,6-dimethoxyphenyl)-1-(2-hydroxy-4-methoxyphenyl)prop-2-en-1-one	$C_{18}H_{18}O_5$
39	(E)-3-(2,4-dimethoxyphenyl)-1-(2-hydroxy-4-methoxyphenyl)prop-2-en-1-one	$C_{18}H_{18}O_5$
40	(E)-3-(2,6-dimethoxyphenyl)-1-(2-hydroxy-4,6-dimethoxyphenyl)prop-2-en-1-one	$C_{19}H_{20}O_6$
41	(E)-3-(3,5-dimethoxyphenyl)-1-(2-hydroxy-4,6-dimethoxyphenyl)prop-2-en-1-one	$C_{19}H_{20}O_6$
42	(E)-1-(2-hydroxy-4,6-dimethoxyphenyl)-3-phenylprop-2-en-1-one	$C_{17}H_{16}O_4$
43	(E)-1-(2-hydroxy-4,6-dimethoxyphenyl)-3-(2-methoxyphenyl)prop-2-en-1-one	$C_{18}H_{18}O_5$

44	(E)-1-(2-hydroxy-4,6-dimethoxyphenyl)-3-(3-methoxyphenyl)prop-2-en-1-one	$C_{18}H_{18}O_5$
45	(E)-3-(2,4-dimethoxyphenyl)-1-(2-hydroxy-4,6-dimethoxyphenyl)prop-2-en-1-one	$C_{19}H_{20}O_6$
46	(E)-1-(2-hydroxy-4,6-dimethoxyphenyl)-3-(2,3,4-trimethoxyphenyl)prop-2-en-1-one	$C_{20}H_{22}O_7$
47	(E)-1-(2-hydroxy-4,6-dimethoxyphenyl)-3-(2,4,5-trimethoxyphenyl)prop-2-en-1-one	$C_{20}H_{22}O_7$
48	(E)-3-(2,4-dimethoxyphenyl)-1-(1-methyl-1H-indol-3-yl)prop-2-en-1-one	$C_{20}H_{19}NO_3$
49	(E)-1-(1-methyl-1H-indol-3-yl)-3-(3,4,5-trimethoxyphenyl)prop-2-en-1-one	$C_{21}H_{21}NO_4$
50	(E)-1-(1-methyl-1H-indol-3-yl)-3-(2,4,6-trimethoxyphenyl)prop-2-en-1-one	$C_{21}H_{21}NO_4$
51	(E)-3-(2,6-dimethoxyphenyl)-1-(1-methyl-1H-indol-3-yl)prop-2-en-1-one	$C_{20}H_{19}NO_3$
52	(E)-1-(5-methoxy-1-methyl-1H-indol-3-yl)-3-(2,4,6-trimethoxyphenyl)prop-2-en-1-one	$C_{22}H_{23}NO_5$
53	(E)-1-(2,4-dimethoxyphenyl)-3-(1-methyl-1H-indol-3-yl)prop-2-en-1-one	$C_{20}H_{19}NO_3$
54	(E)-1-(4-methoxyphenyl)-3-(1-methyl-1H-indol-3-yl)prop-2-en-1-one	$C_{19}H_{17}NO_2$
55	(E)-3-(1-methyl-1H-indol-3-yl)-1-(2,4,6-trimethoxyphenyl)prop-2-en-1-one	$C_{21}H_{21}NO_4$
56	(E)-1-(2,6-dimethoxyphenyl)-3-(1-methyl-1H-indol-3-yl)prop-2-en-1-one	$C_{20}H_{19}NO_3$
57	(E)-1,3-bis(1-methyl-1H-indol-3-yl)prop-2-en-1-one	$C_{21}H_{18}N_2O$
58	(E)-3-(3-chloro-4,5-dimethoxyphenyl)-1-(naphthalen-2-yl)prop-2-en-1-one	$C_{21}H_{17}ClO_3$
59	(E)-3-(3-bromo-4,5-dimethoxyphenyl)-1-(naphthalen-2-yl)prop-2-en-1-one	$C_{21}H_{17}BrO_3$
60	(E)-3-(3-iodo-4,5-dimethoxyphenyl)-1-(naphthalen-2-yl)prop-2-en-1-one	$C_{21}H_{17}IO_3$
61	(E)-1-(naphthalen-2-yl)-3-(3,4,5-trimethoxyphenyl)prop-2-en-1-one	$C_{22}H_{20}O_4$
62	(E)-3-(3,4-dimethoxyphenyl)-1-(3-(quinazolin-4-ylamino)phenyl)prop-2-en-1-one	$C_{25}H_{21}N_3O_3$
63	(E)-3-(4-methoxyphenyl)-1-(3-(quinazolin-4-ylamino)phenyl)prop-2-en-1-one	$C_{24}H_{19}N_3O_2$
64	(E)-3-(3,4-dimethoxyphenyl)-1-(3-((6,7-dimethoxyquinazolin-4-yl)amino)phenyl)prop-2-en-1-one	$C_{27}H_{25}N_3O_5$
65	(E)-1-(3-((6,7-dimethoxyquinazolin-4-yl)amino)phenyl)-3-(4-methoxyphenyl)prop-2-en-1-one	$C_{26}H_{23}N_3O_4$
66	(E)-3-(3,4-dimethoxyphenyl)-1-(3-((2-	$C_{31}H_{25}N_3O_3$

	phenylquinazolin-4-yl)amino)phenyl)prop-2-en-1-one	
67	(E)-3-(4-methoxyphenyl)-1-(3-((2-phenylquinazolin-4-yl)amino)phenyl)prop-2-en-1-one	$C_{30}H_{23}N_3O_2$
68	(E)-3-(3-methoxyphenyl)-1-(3-((2-phenylquinazolin-4-yl)amino)phenyl)prop-2-en-1-one	$C_{30}H_{23}N_3O_2$
69	(E)-3-(3,4-dimethoxyphenyl)-1-(3-((2-(3,4-dimethoxyphenyl)quinazolin-4-yl)amino)phenyl)prop-2-en-1-one	$C_{33}H_{29}N_3O_5$
70	(E)-1-(3-((2-(3,4-dimethoxyphenyl)quinazolin-4-yl)amino)phenyl)-3-(4-methoxyphenyl)prop-2-en-1-one	$C_{32}H_{27}N_3O_4$
71	(E)-1-(3-((2-(3,4-dimethoxyphenyl)quinazolin-4-yl)amino)phenyl)-3-(3-methoxyphenyl)prop-2-en-1-one	$C_{32}H_{27}N_3O_4$
72	(E)-3-(3,4-dimethoxyphenyl)-1-(4-(quinazolin-4-ylamino)phenyl)prop-2-en-1-one	$C_{25}H_{21}N_3O_3$
73	(E)-3-(3-methoxyphenyl)-1-(4-(quinazolin-4-ylamino)phenyl)prop-2-en-1-one	$C_{24}H_{19}N_3O_2$
74	(E)-3-(3,4-dimethoxyphenyl)-1-(4-((6,7-dimethoxyquinazolin-4-yl)amino)phenyl)prop-2-en-1-one	$C_{27}H_{25}N_3O_5$
75	(E)-1-(4-((6,7-dimethoxyquinazolin-4-yl)amino)phenyl)-3-(3-methoxyphenyl)prop-2-en-1-one	$C_{26}H_{23}N_3O_4$
76	(E)-3-(3,4-dimethoxyphenyl)-1-(4-((2-phenylquinazolin-4-yl)amino)phenyl)prop-2-en-1-one	$C_{31}H_{25}N_3O_3$
77	(E)-3-(3-methoxyphenyl)-1-(4-((2-phenylquinazolin-4-yl)amino)phenyl)prop-2-en-1-one	$C_{30}H_{23}N_3O_2$
78	(E)-3-(4-methoxyphenyl)-1-(4-((2-phenylquinazolin-4-yl)amino)phenyl)prop-2-en-1-one	$C_{30}H_{23}N_3O_2$
79	(E)-3-phenyl-1-(4-((2-phenylquinazolin-4-yl)amino)phenyl)prop-2-en-1-one	$C_{29}H_{21}N_3O$
80	(E)-3-(3,4-dimethoxyphenyl)-1-(4-((2-(3,4-dimethoxyphenyl)quinazolin-4-yl)amino)phenyl)prop-2-en-1-one	$C_{33}H_{29}N_3O_5$
81	(E)-1-(4-((2-(3,4-dimethoxyphenyl)quinazolin-4-yl)amino)phenyl)-3-(3-methoxyphenyl)prop-2-en-1-one	$C_{32}H_{27}N_3O_4$
82	(E)-1-(4-((2-(3,4-dimethoxyphenyl)quinazolin-4-yl)amino)phenyl)-3-(4-methoxyphenyl)prop-2-en-1-one	$C_{32}H_{27}N_3O_4$
83	(E)-1-(4-((2-(3,4-dimethoxyphenyl)quinazolin-4-yl)amino)phenyl)-3-phenylprop-2-en-1-one	$C_{31}H_{25}N_3O_3$
84	(E)-1-(4-methoxyphenyl)-3-(quinoxalin-6-yl)prop-2-en-1-one	$C_{18}H_{14}N_2O_2$
85	(E)-1-(2,5-dimethoxyphenyl)-3-(quinoxalin-6-yl)prop-2-en-1-one	$C_{19}H_{16}N_2O_3$
86	(E)-1-(3,4-dimethoxyphenyl)-3-(quinoxalin-6-yl)prop-2-en-1-one	$C_{19}H_{16}N_2O_3$
87	(E)-1-(2,4-dimethoxyphenyl)-3-(quinoxalin-6-yl)prop-2-en-1-one	$C_{19}H_{16}N_2O_3$

88	(E)-3-(quinoxalin-6-yl)-1-(3,4,5-trimethoxyphenyl)prop-2-en-1-one	$C_{20}H_{18}N_2O_4$
89	(E)-1-(4-hydroxy-3-methoxyphenyl)-3-(quinoxalin-6-yl)prop-2-en-1-one	$C_{18}H_{14}N_2O_3$
90	(E)-3-(quinoxalin-6-yl)-1-(2,4,5-trimethoxyphenyl)prop-2-en-1-one	$C_{20}H_{18}N_2O_4$
91	(E)-1-(3,5-dimethoxyphenyl)-3-(quinoxalin-6-yl)prop-2-en-1-one	$C_{19}H_{16}N_2O_3$
92	(E)-1-(2-methoxyphenyl)-3-(quinoxalin-6-yl)prop-2-en-1-one	$C_{18}H_{14}N_2O_2$
93	(E)-1-(4-hydroxy-3,5-dimethoxyphenyl)-3-(quinoxalin-6-yl)prop-2-en-1-one	$C_{19}H_{16}N_2O_4$
94	(E)-1-(3-methoxyphenyl)-3-(quinoxalin-6-yl)prop-2-en-1-one	$C_{18}H_{14}N_2O_2$
95	(E)-3-(quinoxalin-6-yl)-1-(2,3,4-trimethoxyphenyl)prop-2-en-1-one	$C_{20}H_{18}N_2O_4$
96	(E)-N-(3-(3-(3,4-dimethoxyphenyl)acryloyl)phenyl)benzamide	$C_{24}H_{21}NO_4$
97	(E)-N-(3-(3-(3,4-dimethoxyphenyl)acryloyl)phenyl)-4-nitrobenzamide	$C_{24}H_{20}N_2O_6$
98	(E)-3-bromo-N-(3-(3-(3,4-dimethoxyphenyl)acryloyl)phenyl)benzamide	$C_{24}H_{20}BrNO_4$
99	(E)-N-(4-(3-(3,4-dimethoxyphenyl)acryloyl)phenyl)benzamide	$C_{24}H_{21}NO_4$
100	(E)-N-(4-(3-(3,4-dimethoxyphenyl)acryloyl)phenyl)-2-nitrobenzamide	$C_{24}H_{20}N_2O_6$
101	(E)-N-(4-(3-(3,4-dimethoxyphenyl)acryloyl)phenyl)-3-nitrobenzamide	$C_{24}H_{20}N_2O_6$
102	(E)-N-(4-(3-(3,4-dimethoxyphenyl)acryloyl)phenyl)-4-nitrobenzamide	$C_{24}H_{20}N_2O_6$
103	(E)-3-bromo-N-(4-(3-(3,4-dimethoxyphenyl)acryloyl)phenyl)benzamide	$C_{24}H_{20}BrNO_4$
104	(E)-4-bromo-N-(4-(3-(3,4-dimethoxyphenyl)acryloyl)phenyl)benzamide	$C_{24}H_{20}BrNO_4$
105	(E)-3-chloro-N-(4-(3-(3,4-dimethoxyphenyl)acryloyl)phenyl)benzamide	$C_{24}H_{20}ClNO_4$
106	(E)-4-chloro-N-(4-(3-(3,4-dimethoxyphenyl)acryloyl)phenyl)benzamide	$C_{24}H_{20}ClNO_4$
107	(E)-3,4-dichloro-N-(4-(3-(3,4-dimethoxyphenyl)acryloyl)phenyl)benzamide	$C_{24}H_{19}Cl_2NO_4$
108	(E)-N-(4-(3-(3,4-dimethoxyphenyl)acryloyl)phenyl)-2-fluorobenzamide	$C_{24}H_{20}FNO_4$
109	(E)-4-cyano-N-(4-(3-(3,4-dimethoxyphenyl)acryloyl)phenyl)benzamide	$C_{25}H_{20}N_2O_4$
110	(E)-N-(4-(3-(3,4-dimethoxyphenyl)acryloyl)phenyl)-4-	$C_{25}H_{20}F_3NO_4$

	(trifluoromethyl)benzamide	
111	(E)-N-(4-(3-(3,4-dimethoxyphenyl)acryloyl)phenyl)-2-methoxybenzamide	$C_{25}H_{23}NO_5$
112	(E)-N-(4-(3-(3,4-dimethoxyphenyl)acryloyl)phenyl)-3-methoxybenzamide	$C_{25}H_{23}NO_5$
113	(E)-N-(4-(3-(3,4-dimethoxyphenyl)acryloyl)phenyl)-4-methoxybenzamide	$C_{25}H_{23}NO_5$
114	(E)-N-(4-(3-(3,4-dimethoxyphenyl)acryloyl)phenyl)-3,4-dimethoxybenzamide	$C_{26}H_{25}NO_6$
115	(E)-N-(4-(3-(3,4-dimethoxyphenyl)acryloyl)phenyl)-3,4,5-trimethoxybenzamide	$C_{27}H_{27}NO_7$
116	(E)-N-(2-(3-(3,4-dimethoxyphenyl)acryloyl)phenyl)benzamide	$C_{24}H_{21}NO_4$
117	(E)-3-chloro-N-(2-(3-(3,4-dimethoxyphenyl)acryloyl)phenyl)benzamide	$C_{24}H_{20}ClNO_4$
118	(E)-4-chloro-N-(2-(3-(3,4-dimethoxyphenyl)acryloyl)phenyl)benzamide	$C_{24}H_{20}ClNO_4$
119	(E)-N-(2-(3-(3,4-dimethoxyphenyl)acryloyl)phenyl)-2-nitrobenzamide	$C_{24}H_{20}N_2O_6$
120	(E)-N-(2-(3-(3,4-dimethoxyphenyl)acryloyl)phenyl)-3-nitrobenzamide	$C_{24}H_{20}N_2O_6$
121	(E)-N-(2-(3-(3,4-dimethoxyphenyl)acryloyl)phenyl)-2-methoxybenzamide	$C_{25}H_{23}NO_5$
122	(E)-N-(2-(3-(3,4-dimethoxyphenyl)acryloyl)phenyl)-3-methoxybenzamide	$C_{25}H_{23}NO_5$
123	(E)-N-(2-(3-(3,4-dimethoxyphenyl)acryloyl)phenyl)-3-(quinolin-3-yl)benzamide	$C_{33}H_{26}N_2O_4$
124	(E)-N-(2-(3-(3,4-dimethoxyphenyl)acryloyl)phenyl)-3-(naphthalen-1-yl)benzamide	$C_{34}H_{27}NO_4$
125	(E)-N-(2-(3-(3,4-dimethoxyphenyl)acryloyl)phenyl)-3-(pyridin-3-yl)benzamide	$C_{29}H_{24}N_2O_4$
126	(E)-N-(2-(3-(3,4-dimethoxyphenyl)acryloyl)phenyl)-3-(7-methoxy-2-oxo-2H-chromen-5-yl)benzamide	$C_{34}H_{27}NO_7$
127	(E)-N-(2-(3-(3,4-dimethoxyphenyl)acryloyl)phenyl)-2-(thiophen-2-yl)benzamide	$C_{28}H_{23}NO_4S$
128	(E)-3-chloro-N-(2-(3-(3-chlorophenyl)acryloyl)phenyl)benzamide	$C_{22}H_{15}Cl_2NO_2$
129	(E)-N-(2-(3-(3-chlorophenyl)acryloyl)phenyl)-3,4-dimethoxybenzamide	$C_{24}H_{20}ClNO_4$
130	(E)-N-(2-(3-(4-fluoro-3-methoxyphenyl)acryloyl)phenyl)-2-(thiophen-2-yl)benzamide	$C_{27}H_{20}FNO_3S$
131	(E)-3-chloro-N-(2-(3-(4-fluoro-3-methoxyphenyl)acryloyl)phenyl)benzamide	$C_{23}H_{17}ClFNO_3$
132	(E)-N-(3-(3-(4-(2-(6,7-dimethoxy-3,4-	$C_{38}H_{35}N_3O_5$

	dihydroisoquinolin-2(1H)-yl)ethoxy)phenyl)acryloyl)phenyl)quinoline-2-carboxamide	
133	(E)-N-(3-(3-(4-(2-(6-methoxy-7-(2-methoxyethyl)-3,4-dihydroisoquinolin-2(1H)-yl)ethoxy)phenyl)acryloyl)phenyl)quinoline-2-carboxamide	$C_{40}H_{39}N_3O_5$
134	(E)-N-(3-(3-(4-(2-(6-methoxy-7-(2-(2-(2-methoxyethoxy)ethoxy)ethyl)-3,4-dihydroisoquinolin-2(1H)-yl)ethoxy)phenyl)acryloyl)phenyl)quinoline-2-carboxamide	$C_{44}H_{47}N_3O_7$
135	(E)-N-(5-(3-(4-(2-(6,7-dimethoxy-3,4-dihydroisoquinolin-2(1H)-yl)ethoxy)phenyl)acryloyl)-2-methylphenyl)quinoline-2-carboxamide	$C_{39}H_{37}N_3O_5$
136	(E)-N-(5-(3-(4-(2-(6-methoxy-7-(2-methoxyethyl)-3,4-dihydroisoquinolin-2(1H)-yl)ethoxy)phenyl)acryloyl)-2-methylphenyl)quinoline-2-carboxamide	$C_{41}H_{41}N_3O_5$
137	(E)-N-(5-(3-(4-(2-(6-methoxy-7-(2-(2-(2-methoxyethoxy)ethoxy)ethyl)-3,4-dihydroisoquinolin-2(1H)-yl)ethoxy)phenyl)acryloyl)-2-methylphenyl)quinoline-2-carboxamide	$C_{45}H_{49}N_3O_7$
138	(E)-1-(2-(allyloxy)-4-methoxyphenyl)-3-(4-methoxyphenyl)prop-2-en-1-one	$C_{20}H_{20}O_4$
139	(E)-3-(1H-indol-5-yl)-1-(5-methoxy-2,2-dimethyl-2H-chromen-8-yl)prop-2-en-1-one	$C_{23}H_{21}NO_3$
140	(E)-2-(2-methoxy-5-(3-(5-methoxy-2,2-dimethyl-2H-chromen-8-yl)-3-oxoprop-1-en-1-yl)phenoxy)-N-(2-methoxyphenyl)acetamide	$C_{31}H_{31}NO_7$
141	(E)-N-(2-chlorophenyl)-2-(2-methoxy-5-(3-oxo-3-(3,4,5-trimethoxyphenyl)prop-1-en-1-yl)phenoxy)acetamide	$C_{27}H_{26}ClNO_7$
142	(E)-3-(3-hydroxy-4-methoxyphenyl)-1-(2-methoxynaphthalen-1-yl)prop-2-en-1-one	$C_{21}H_{18}O_4$
143	(E)-3-(4-bromophenyl)-1-(2-methoxynaphthalen-1-yl)prop-2-en-1-one	$C_{20}H_{15}BrO_2$
144	(E)-1-(2-methoxynaphthalen-1-yl)-3-(2,3,4-trimethoxyphenyl)prop-2-en-1-one	$C_{23}H_{22}O_5$
145	(E)-3-(2-bromophenyl)-1-(2-methoxynaphthalen-1-yl)prop-2-en-1-one	$C_{20}H_{15}BrO_2$
146	(E)-1-(2-methoxynaphthalen-1-yl)-3-(naphthalen-1-yl)prop-2-en-1-one	$C_{24}H_{18}O_2$
147	(E)-1-(2-methoxynaphthalen-1-yl)-3-(naphthalen-2-yl)prop-2-en-1-one	$C_{24}H_{18}O_2$
148	(E)-3-(3-bromo-4-methoxyphenyl)-1-(2-methoxynaphthalen-1-yl)prop-2-en-1-one	$C_{21}H_{17}BrO_3$
149	(E)-1-(2-methoxynaphthalen-1-yl)-3-(3-	$C_{21}H_{18}O_3$

	methoxyphenyl)prop-2-en-1-one	
150	(E)-3-(4-methoxy-3-nitrophenyl)-1-(2-methoxynaphthalen-1-yl)prop-2-en-1-one	$C_{21}H_{17}NO_5$
151	(E)-1-(2-methoxynaphthalen-1-yl)-3-(3-(thiophen-2-yl)phenyl)prop-2-en-1-one	$C_{24}H_{18}O_2S$
152	(E)-3-(4-chlorophenyl)-1-(2-methoxynaphthalen-1-yl)prop-2-en-1-one	$C_{20}H_{15}ClO_2$
153	(E)-1-(2-methoxynaphthalen-1-yl)-3-(2,4,5-trimethoxyphenyl)prop-2-en-1-one	$C_{23}H_{22}O_5$
154	(E)-3-(2-fluorophenyl)-1-(2-methoxynaphthalen-1-yl)prop-2-en-1-one	$C_{20}H_{15}FO_2$
155	(E)-1-(2-methoxynaphthalen-1-yl)-3-phenylprop-2-en-1-one	$C_{20}H_{16}O_2$
156	(E)-3-(3-fluorophenyl)-1-(2-methoxynaphthalen-1-yl)prop-2-en-1-one	$C_{20}H_{15}FO_2$
157	(E)-1-(2-methoxynaphthalen-1-yl)-3-(3,4,5-trimethoxyphenyl)prop-2-en-1-one	$C_{23}H_{22}O_5$
158	(E)-1-(2-methoxynaphthalen-1-yl)-3-(2-methoxyphenyl)prop-2-en-1-one	$C_{21}H_{18}O_3$
159	(E)-3-(4-(dimethylamino)phenyl)-1-(2-methoxynaphthalen-1-yl)prop-2-en-1-one	$C_{22}H_{21}NO_2$
160	(E)-3-(4-(diethylamino)phenyl)-1-(2-methoxynaphthalen-1-yl)prop-2-en-1-one	$C_{24}H_{25}NO_2$
161	(E)-3-(3-amino-4-methoxyphenyl)-1-(2-methoxynaphthalen-1-yl)prop-2-en-1-one	$C_{21}H_{19}NO_3$
162	(E)-3-(4-chlorophenyl)-1-(3-(3,4,5-trimethoxyphenoxy)phenyl)prop-2-en-1-one	$C_{24}H_{21}ClO_5$
163	(E)-3-(4-methoxyphenyl)-1-(3-(3,4,5-trimethoxyphenoxy)phenyl)prop-2-en-1-one	$C_{25}H_{24}O_6$
164	(E)-3-(4-(diethylamino)phenyl)-1-(3-(3,4,5-trimethoxyphenoxy)phenyl)prop-2-en-1-one	$C_{28}H_{31}NO_5$
165	(E)-3-(4-(dimethylamino)phenyl)-1-(3-(3,4,5-trimethoxyphenoxy)phenyl)prop-2-en-1-one	$C_{26}H_{27}NO_5$
166	(E)-1-(3-(3,4,5-trimethoxyphenoxy)phenyl)-3-(3,4,5-trimethoxyphenyl)prop-2-en-1-one	$C_{27}H_{28}O_8$
167	(E)-3-phenyl-1-(3-(3,4,5-trimethoxyphenoxy)phenyl)prop-2-en-1-one	$C_{24}H_{22}O_5$
168	(E)-3-(2-fluorophenyl)-1-(3-(3,4,5-trimethoxyphenoxy)phenyl)prop-2-en-1-one	$C_{24}H_{21}FO_5$
169	(E)-3-(naphthalen-1-yl)-1-(3-(3,4,5-trimethoxyphenoxy)phenyl)prop-2-en-1-one	$C_{28}H_{24}O_5$
170	(E)-3-(4-bromophenyl)-1-(3-(3,4,5-trimethoxyphenoxy)phenyl)prop-2-en-1-one	$C_{24}H_{21}BrO_5$
171	(E)-3-(4-fluorophenyl)-1-(3-(3,4,5-trimethoxyphenoxy)phenyl)prop-2-en-1-one	$C_{24}H_{21}FO_5$

172	(E)-3-(3-fluorophenyl)-1-(3-(3,4,5-trimethoxyphenoxy)phenyl)prop-2-en-1-one	$C_{24}H_{21}FO_5$
173	(E)-1-(3-(3,4,5-trimethoxyphenoxy)phenyl)-3-(2,3,4-trimethoxyphenyl)prop-2-en-1-one	$C_{27}H_{28}O_8$
174	(E)-3-(4-methoxy-3-nitrophenyl)-1-(3-(3,4,5-trimethoxyphenoxy)phenyl)prop-2-en-1-one	$C_{25}H_{23}NO_8$
175	(E)-3-(naphthalen-2-yl)-1-(3-(3,4,5-trimethoxyphenoxy)phenyl)prop-2-en-1-one	$C_{28}H_{24}O_5$
176	(E)-3-(3-chlorophenyl)-1-(3-(3,4,5-trimethoxyphenoxy)phenyl)prop-2-en-1-one	$C_{24}H_{21}ClO_5$
177	(E)-3-(3-amino-4-methoxyphenyl)-1-(3-(3,4,5-trimethoxyphenoxy)phenyl)prop-2-en-1-one	$C_{25}H_{25}NO_6$
178	(E)-3-(5-methoxy-2-methyl-1H-indol-3-yl)-1-(pyridin-4-yl)prop-2-en-1-one	$C_{18}H_{16}N_2O_2$
179	(E)-3-(3-hydroxy-4-methoxyphenyl)-1-(7-methoxybenzo[d][1,3]dioxol-5-yl)prop-2-en-1-one	$C_{18}H_{16}O_6$
180	(E)-3-(3-fluoro-4-methoxyphenyl)-1-(7-methoxybenzo[d][1,3]dioxol-5-yl)prop-2-en-1-one	$C_{18}H_{15}FO_5$
181	(E)-1-(7-methoxybenzo[d][1,3]dioxol-5-yl)-3-(6-methoxypyridin-3-yl)prop-2-en-1-one	$C_{17}H_{15}NO_5$
182	(E)-3-(4-methoxy-3-nitrophenyl)-1-(7-methoxybenzo[d][1,3]dioxol-5-yl)prop-2-en-1-one	$C_{18}H_{15}NO_7$
183	(E)-3-(3-amino-4-methoxyphenyl)-1-(7-methoxybenzo[d][1,3]dioxol-5-yl)prop-2-en-1-one	$C_{18}H_{17}NO_5$
184	(E)-3-(3-hydroxy-4-methoxyphenyl)-1-(7-methoxybenzo[d][1,3]dioxol-5-yl)-2-methylprop-2-en-1-one	$C_{19}H_{18}O_6$
185	(E)-3-(3-amino-4-methoxyphenyl)-1-(7-methoxybenzo[d][1,3]dioxol-5-yl)-2-methylprop-2-en-1-one	$C_{19}H_{19}NO_5$
186	(E)-O-((2-methoxy-5-(3-(7-methoxybenzo[d][1,3]dioxol-5-yl)-2-methyl-3-oxoprop-1-en-1-yl)phenyl)amino)serine	$C_{22}H_{24}N_2O_8$
187	(E)-2-amino-5-(((5-(((2-methoxy-5-(3-(7-methoxybenzo[d][1,3]dioxol-5-yl)-2-methyl-3-oxoprop-1-en-1-yl)phenyl)amino)oxy)pyrrolidine-2-carbonyl)oxy)amino)pentanoic acid	$C_{29}H_{36}N_4O_{10}$
188	(E)-3-(3-(2-methoxyphenyl)-3-oxoprop-1-en-1-yl)quinolin-2(1H)-one	$C_{19}H_{15}NO_3$
189	(E)-6-methoxy-3-(3-(2-methoxyphenyl)-3-oxoprop-1-en-1-yl)quinolin-2(1H)-one	$C_{20}H_{17}NO_4$
190	(E)-1-(4-methoxyphenyl)-3-(4-(4-(piperidin-1-yl)butoxy)phenyl)prop-2-en-1-one	$C_{25}H_{31}NO_3$
191	(E)-1-(2-hydroxyphenyl)-3-(4-(4-(piperidin-1-yl)butoxy)phenyl)prop-2-en-1-one	$C_{24}H_{29}NO_3$

192	(E)-1-(4-bromophenyl)-3-(4-(4-(piperidin-1-yl)butoxy)phenyl)prop-2-en-1-one	$C_{24}H_{28}BrNO_2$
193	(E)-1-phenyl-3-(4-(4-(piperidin-1-yl)butoxy)phenyl)prop-2-en-1-one	$C_{24}H_{29}NO_2$
194	(E)-1-(4-bromophenyl)-3-(4-(4-(piperidin-1-yl)butoxy)phenyl)prop-2-en-1-one	$C_{24}H_{28}BrNO_2$
195	(E)-1-(4-chlorophenyl)-3-(4-(4-(piperidin-1-yl)butoxy)phenyl)prop-2-en-1-one	$C_{24}H_{28}ClNO_2$
196	(E)-1-(2,4-dichlorophenyl)-3-(4-(4-(piperidin-1-yl)butoxy)phenyl)prop-2-en-1-one	$C_{24}H_{27}Cl_2NO_2$
197	(E)-1-(4-methoxyphenyl)-3-(4-(2-morpholinoethoxy)phenyl)prop-2-en-1-one	$C_{22}H_{25}NO_4$
198	(E)-1-(4-bromophenyl)-3-(4-(2-morpholinoethoxy)phenyl)prop-2-en-1-one	$C_{21}H_{22}BrNO_3$
199	(E)-1-(2,4-dichlorophenyl)-3-(4-(2-morpholinoethoxy)phenyl)prop-2-en-1-one	$C_{21}H_{21}Cl_2NO_3$
200	(E)-3-(4-(2-morpholinoethoxy)phenyl)-1-(4-(trifluoromethyl)phenyl)prop-2-en-1-one	$C_{22}H_{22}F_3NO_3$
201	(E)-1-(4-methoxyphenyl)-3-(4-(3-morpholinopropoxy)phenyl)prop-2-en-1-one	$C_{23}H_{27}NO_4$
202	(E)-3-(4-(3-(2,6-dimethylmorpholino)propoxy)phenyl)-1-(4-methoxyphenyl)prop-2-en-1-one	$C_{25}H_{31}NO_4$
203	(E)-3-(4-(3-(2,6-dimethylmorpholino)propoxy)phenyl)-1-(2-methoxyphenyl)prop-2-en-1-one	$C_{25}H_{31}NO_4$
204	(E)-1-(2,4-dichlorophenyl)-3-(4-(3-(2,6-dimethylmorpholino)propoxy)phenyl)prop-2-en-1-one	$C_{24}H_{27}Cl_2NO_3$
205	(E)-3-(4-(3-(2,6-dimethylmorpholino)propoxy)phenyl)-1-(p-tolyl)prop-2-en-1-one	$C_{25}H_{31}NO_3$
206	(E)-3-(4-(4-(indolin-2-yl)butoxy)phenyl)-1-(4-methoxyphenyl)prop-2-en-1-one	$C_{28}H_{29}NO_3$
207	(E)-3-(4-(4-(indolin-1-yl)butoxy)phenyl)-1-(4-methoxyphenyl)prop-2-en-1-one	$C_{28}H_{29}NO_3$
208	(E)-3-(4-(4-(3,4-dihydroquinolin-1(2H)-yl)butoxy)phenyl)-1-phenylprop-2-en-1-one	$C_{28}H_{29}NO_2$
209	(E)-3-(4-(4-(3,4-dihydroquinolin-1(2H)-yl)butoxy)phenyl)-1-(4-methoxyphenyl)prop-2-en-1-one	$C_{29}H_{31}NO_3$
210	(E)-1-(4-bromophenyl)-3-(4-(4-(3,4-dihydroquinolin-1(2H)-yl)butoxy)phenyl)prop-2-en-1-one	$C_{28}H_{28}BrNO_2$
211	(E)-1-(4-chlorophenyl)-3-(4-(4-(3,4-dihydroquinolin-1(2H)-yl)butoxy)phenyl)prop-2-en-1-one	$C_{28}H_{28}ClNO_2$
212	(E)-1-(2,4-dichlorophenyl)-3-(4-(4-(3,4-dihydroquinolin-1(2H)-yl)butoxy)phenyl)prop-2-en-1-one	$C_{28}H_{27}Cl_2NO_2$
213	(E)-1-(4-aminophenyl)-3-(4-(4-(3,4-dihydroquinolin-1(2H)-yl)butoxy)phenyl)prop-2-en-1-one	$C_{28}H_{30}N_2O_2$

214	(E)-1-(4-aminophenyl)-3-(4-(4-(3,5-dimethyl-1H-pyrazol-1-yl)butoxy)phenyl)prop-2-en-1-one	$C_{24}H_{27}N_3O_2$
215	(1R)-1-(5,8-dihydroxy-1,4-dioxo-1,2,3,4-tetrahydronaphthalen-2-yl)-4-methylpent-3-en-1-yl 2-(4-((E)-3-oxo-3-phenylprop-1-en-1-yl)phenoxy)acetate	$C_{33}H_{30}O_8$
216	(1R)-1-(5,8-dihydroxy-1,4-dioxo-1,2,3,4-tetrahydronaphthalen-2-yl)-4-methylpent-3-en-1-yl 2-(4-((E)-3-(4-methoxyphenyl)-3-oxoprop-1-en-1-yl)phenoxy)acetate	$C_{34}H_{32}O_9$
217	(1R)-1-(5,8-dihydroxy-1,4-dioxo-1,2,3,4-tetrahydronaphthalen-2-yl)-4-methylpent-3-en-1-yl 2-(4-((E)-3-oxo-3-(3,4,5-trimethoxyphenyl)prop-1-en-1-yl)phenoxy)acetate	$C_{36}H_{36}O_{11}$
218	(1R)-1-(5,8-dihydroxy-1,4-dioxo-1,2,3,4-tetrahydronaphthalen-2-yl)-4-methylpent-3-en-1-yl 2-(4-((E)-3-oxo-3-(4-(trifluoromethyl)phenyl)prop-1-en-1-yl)phenoxy)acetate	$C_{34}H_{29}F_3O_8$
219	(1R)-1-(5,8-dihydroxy-1,4-dioxo-1,2,3,4-tetrahydronaphthalen-2-yl)-4-methylpent-3-en-1-yl 2-(4-((E)-3-oxo-3-(2,3,4-trichlorophenyl)prop-1-en-1-yl)phenoxy)acetate	$C_{33}H_{27}Cl_3O_8$
220	(1R)-1-(5,8-dihydroxy-1,4-dioxo-1,2,3,4-tetrahydronaphthalen-2-yl)-4-methylpent-3-en-1-yl 2-(4-((E)-3-oxo-3-(2-(trifluoromethyl)phenyl)prop-1-en-1-yl)phenoxy)acetate	$C_{34}H_{29}F_3O_8$
221	(1R)-1-(5,8-dihydroxy-1,4-dioxo-1,2,3,4-tetrahydronaphthalen-2-yl)-4-methylpent-3-en-1-yl 2-(4-((E)-3-(2-methoxyphenyl)-3-oxoprop-1-en-1-yl)phenoxy)acetate	$C_{34}H_{32}O_9$
222	(1R)-1-(5,8-dihydroxy-1,4-dioxo-1,2,3,4-tetrahydronaphthalen-2-yl)-4-methylpent-3-en-1-yl 2-(4-((E)-3-oxo-3-(3-(trifluoromethyl)phenyl)prop-1-en-1-yl)phenoxy)acetate	$C_{34}H_{29}F_3O_8$
223	(1R)-1-(5,8-dihydroxy-1,4-dioxo-1,2,3,4-tetrahydronaphthalen-2-yl)-4-methylpent-3-en-1-yl 2-(4-((E)-3-(4-ethylphenyl)-3-oxoprop-1-en-1-yl)phenoxy)acetate	$C_{35}H_{34}O_8$
224	(1R)-1-(5,8-dihydroxy-1,4-dioxo-1,2,3,4-tetrahydronaphthalen-2-yl)-4-methylpent-3-en-1-yl 2-(4-((E)-3-(2-fluorophenyl)-3-oxoprop-1-en-1-yl)phenoxy)acetate	$C_{33}H_{29}FO_8$
225	(1R)-1-(5,8-dihydroxy-1,4-dioxo-1,2,3,4-tetrahydronaphthalen-2-yl)-4-methylpent-3-en-1-yl 2-(4-((E)-3-(3,5-bis(trifluoromethyl)phenyl)-3-oxoprop-1-en-1-yl)phenoxy)acetate	$C_{35}H_{28}F_6O_8$

226	(1R)-1-(5,8-dihydroxy-1,4-dioxo-1,2,3,4-tetrahydronaphthalen-2-yl)-4-methylpent-3-en-1-yl 2-(4-((E)-3-oxo-3-(3-(trifluoromethoxy)phenyl)prop-1-en-1-yl)phenoxy)acetate	$C_{34}H_{29}F_3O_9$
227	(1R)-1-(5,8-dihydroxy-1,4-dioxo-1,2,3,4-tetrahydronaphthalen-2-yl)-4-methylpent-3-en-1-yl 2-(4-((E)-3-(3-methoxyphenyl)-3-oxoprop-1-en-1-yl)phenoxy)acetate	$C_{34}H_{32}O_9$
228	(1R)-1-(5,8-dihydroxy-1,4-dioxo-1,2,3,4-tetrahydronaphthalen-2-yl)-4-methylpent-3-en-1-yl 2-(4-((E)-3-oxo-3-(o-tolyl)prop-1-en-1-yl)phenoxy)acetate	$C_{34}H_{32}O_8$
229	(1R)-1-(5,8-dihydroxy-1,4-dioxo-1,2,3,4-tetrahydronaphthalen-2-yl)-4-methylpent-3-en-1-yl 2-(4-((E)-3-(2-chlorophenyl)-3-oxoprop-1-en-1-yl)phenoxy)acetate	$C_{33}H_{29}ClO_8$
230	(1R)-1-(5,8-dihydroxy-1,4-dioxo-1,2,3,4-tetrahydronaphthalen-2-yl)-4-methylpent-3-en-1-yl 2-(4-((E)-3-(2-bromophenyl)-3-oxoprop-1-en-1-yl)phenoxy)acetate	$C_{33}H_{29}BrO_8$
231	(1R)-1-(5,8-dihydroxy-1,4-dioxo-1,2,3,4-tetrahydronaphthalen-2-yl)-4-methylpent-3-en-1-yl 2-(4-((E)-3-oxo-3-(m-tolyl)prop-1-en-1-yl)phenoxy)acetate	$C_{34}H_{32}O_8$
232	(1R)-1-(5,8-dihydroxy-1,4-dioxo-1,2,3,4-tetrahydronaphthalen-2-yl)-4-methylpent-3-en-1-yl 2-(4-((E)-3-oxo-3-(p-tolyl)prop-1-en-1-yl)phenoxy)acetate	$C_{34}H_{32}O_8$
233	(E)-3-(2,4-dichlorophenyl)-1-(4-((1-vinyl-1H-1,2,3-triazol-4-yl)methoxy)phenyl)prop-2-en-1-one	$C_{20}H_{15}Cl_2N_3O_2$
