# Supplementary material for: Two Important Anticancer Mechanisms of Natural and Synthetic Chalcones
Source: Int J Mol Sci. 2022 Sep 30;23(19):11595. doi: 10.3390/ijms231911595 (PMC9570335; doi:10.3390/ijms231911595)
Supplement: Supplementary file 1 [file ijms-23-11595-s001.zip › Table S3.pdf]

Table S3. Half-maximal inhibitory concentration (IC50) of the most effective chalcones compounds

Com-pound	IC50 (μM) for the cell lines:										
	A549	HeLa	Bel-7402	MCF-7	HCT-8	H460-MX20	BCRP/ ABCG2	MV-4-11	SK-OV-3	KB-C-2	HepG2
8	0.003± 0.001	0.007± 0.004	0.009± 0.002	0.006± 0.003	0.006± 0.002	-	-	-	-	-	-
9	-	-	-	-	-	0.442± 0.144	-	-	-	-	-
40	-	-	-	-	-	-	0.35±0.14	-	-	-	-
41	-	-	-	-	-	-	0.17±0.07	-	-	-	-
44	-	-	-	-	-	-	0.26±0.04	-	-	-	-
50	-	-	-	-	-	-	0.45±0.09	-	-	-	-
51	-	-	-	-	-	-	0.27±0.08	-	-	-	-
52	-	-	-	-	-	-	0.35±0.09	-	-	-	-
58	-	-	-	1.5±0.4	-	-	8.7±1.3	-	-	-	-
59	-	-	-	2.3±0.3	-	-	4.9±0.3	-	-	-	-
60	-	-	-	1.3±0.4	-	-	7.8±1.6	-	-	-	-
61	-	-	-	5.6±1.0	-	-	2.4±0.5	-	-	-	-
62	-	-	-	-	-	-	1.30±0.12	-	-	-	-
63	-	-	-	-	-	-	0.89±0.24	-	-	-	-
64	-	-	-	-	-	-	1.71±0.07	-	-	-	-
65	-	-	-	-	-	-	0.32±0.02	-	-	-	-
66	-	-	-	-	-	-	0.46±0.03	-	-	-	-
67	-	-	-	-	-	-	0.97±0.17	-	-	-	-
68	-	-	-	-	-	-	0.68±0.04	-	-	-	-
69	-	-	-	-	-	-	0.60±0.04	-	-	-	-
70	-	-	-	-	-	-	0.21±0.02	-	-	-	-
71	-	-	-	-	-	-	0.31±0.05	-	-	-	-
72	-	-	-	-	-	-	0.84±0.16	-	-	-	-
73	-	-	-	-	-	-	0.68±0.04	-	-	-	-

74	-	-	-	-	-	-	1.23±0.17	-	-	-	-
75	-	-	-	-	-	-	0.39±0.01	-	-	-	-
76	-	-	-	-	-	-	0.29±0.09	-	-	-	-
77	-	-	-	-	-	-	0.88±0.09	-	-	-	-
78	-	-	-	-	-	-	3.55±0.19	-	-	-	-
79	-	-	-	-	-	-	0.92±0.13	-	-	-	-
80	-	-	-	-	-	-	0.19±0.02	-	-	-	-
81	-	-	-	-	-	-	0.36±0.06	-	-	-	-
82	-	-	-	-	-	-	3.13±0.30	-	-	-	-
83	-	-	-	-	-	-	1.09±0.13	-	-	-	-
84	-	-	-	-	-	-	2.8±0.5	-	-	-	-
85	-	-	-	-	-	-	1.7±0.2	-	-	-	-
86	-	-	-	-	-	-	1.9±0.7	-	-	-	-
87	-	-	-	-	-	-	1.4±1.0	-	-	-	-
88	-	-	-	-	-	-	2.2±1.5	-	-	-	-
89	-	-	-	-	-	-	16.1±1.5	-	-	-	-
90	-	-	-	-	-	-	1.4±1.0	-	-	-	-
91	-	-	-	-	-	-	2.1±1.3	-	-	-	-
92	-	-	-	-	-	-	14.3±3.2	-	-	-	-
93	-	-	-	-	-	-	11.4±1.5	-	-	-	-
94	-	-	-	-	-	-	5.0±0.6	-	-	-	-
95	-	-	-	-	-	-	11.5±0.4	-	-	-	-
96	-	-	-	-	-	-	2.18±0.13	-	-	-	-
97	-	-	-	-	-	-	>20	-	-	-	-
98	-	-	-	-	-	-	2.99±0.14	-	-	-	-
99	-	-	-	-	-	-	1.30±0.17	-	-	-	-
100	-	-	-	-	-	-	2.98±0.40	-	-	-	-
101	-	-	-	-	-	-	2.82±0.19	-	-	-	-
102	-	-	-	-	-	-	>20	-	-	-	-

103	-	-	-	-	-	-	1.74±0.04	-	-	-	-
104	-	-	-	-	-	-	2.94±0.16	-	-	-	-
105	-	-	-	-	-	-	2.39±0.28	-	-	-	-
106	-	-	-	-	-	-	1.87±0.10	-	-	-	-
107	-	-	-	-	-	-	2.95±0.20	-	-	-	-
108	-	-	-	-	-	-	4.93±0.53	-	-	-	-
109	-	-	-	-	-	-	2.80±0.16	-	-	-	-
110	-	-	-	-	-	-	15.3±3.5	-	-	-	-
111	-	-	-	-	-	-	2.48±0.07	-	-	-	-
112	-	-	-	-	-	-	2.18±0.14	-	-	-	-
113	-	-	-	-	-	-	6.61±0.29	-	-	-	-
114	-	-	-	-	-	-	3.15±0.20	-	-	-	-
115	-	-	-	-	-	-	3.04±0.32	-	-	-	-
116	-	-	-	-	-	-	0.982± 0.149	-	-	-	-
117	-	-	-	-	-	-	0.767± 0.011	-	-	-	-
118	-	-	-	-	-	-	1.86±0.16	-	-	-	-
119	-	-	-	-	-	-	>20	-	-	-	-
120	-	-	-	-	-	-	17.4±6.4	-	-	-	-
121	-	-	-	-	-	-	1.23±0.06	-	-	-	-
122	-	-	-	-	-	-	1.12±0.11	-	-	-	-
123	-	-	-	-	-	-	0.971± 0.084	-	-	-	-
124	-	-	-	-	-	-	1.08±0.07	-	-	-	-
125	-	-	-	-	-	-	2.01±0.09	-	-	-	-
126	-	-	-	-	-	-	>20	-	-	-	-
127	-	-	-	-	-	-	0.600± 0.010	-	-	-	-
128	-	-	-	-	-	-	>20	-	-	-	-
129	-	-	-	-	-	-	3.32±0.18	-	-	-	-

130	-	-	-	-	-	-	3.37±0.05	-	-	-	-
131	-	-	-	-	-	-	10.6±0.6	-	-	-	-
132	-	-	-	4.08±0.29	-	-	-	-	-	-	-
133	-	-	-	2.13±0.47	-	-	-	-	-	-	-
134	-	-	-	2.83±0.30	-	-	-	-	-	-	-
135	-	-	-	0.88±0.02	-	-	-	-	-	-	-
136	-	-	-	1.55±0.36	-	-	-	-	-	-	-
137	-	-	-	2.72±0.54	-	-	-	-	-	-	-
138	-	-	-	-	-	-	-	5.7	-	-	-
140	-	-	-	-	-	-	-	-	14.56± 1.08	-	11.82± 1.17
141	-	-	-	-	-	-	-	-	6.28±0.79	-	4.06±1.21
142	-	-	-	1.42±0.15	-	-	-	-	-	-	-
143	-	-	-	4.10±0.25	-	-	-	-	-	-	-
144	-	-	-	>10	-	-	-	-	-	-	-
145	-	-	-	7.63±0.45	-	-	-	-	-	-	-
146	-	-	-	7.13±0.33	-	-	-	-	-	-	-
147	-	-	-	6.87±0.26	-	-	-	-	-	-	-
148	-	-	-	7.11±0.40	-	-	-	-	-	-	-
149	-	-	-	6.67±0.25	-	-	-	-	-	-	-
150	-	-	-	7.82±0.35	-	-	-	-	-	-	-
151	-	-	-	>10	-	-	-	-	-	-	-
152	-	-	-	4.74±0.19	-	-	-	-	-	-	-
153	-	-	-	7.68±0.32	-	-	-	-	-	-	-
154	-	-	-	6.66±0.27	-	-	-	-	-	-	-
155	-	-	-	7.62±0.36	-	-	-	-	-	-	-
156	-	-	-	7.11±0.47	-	-	-	-	-	-	-
157	-	-	-	7.23±0.38	-	-	-	-	-	-	-
158	-	-	-	7.67±0.39	-	-	-	-	-	-	-
159	-	-	-	8.91±0.52	-	-	-	-	-	-	-

160	-	-	-	7.62±0.30	-	-	-	-	-	-	-
161	-	-	-	2.75±0.26	-	-	-	-	-	-	-
162	-	-	-	4.39±0.26	-	-	-	-	-	-	5.13±0.31
163	-	-	-	3.44±0.19	-	-	-	-	-	-	4.64±0.23
164	-	-	-	>10	-	-	-	-	-	-	>10
165	-	-	-	>10	-	-	-	-	-	-	>10
166	-	-	-	6.13±0.32	-	-	-	-	-	-	7.61±0.49
167	-	-	-	6.39±0.41	-	-	-	-	-	-	>10
168	-	-	-	5.09±0.26	-	-	-	-	-	-	7.68±0.30
169	-	-	-	6.63±0.30	-	-	-	-	-	-	7.11±0.34
170	-	-	-	4.99±0.20	-	-	-	-	-	-	6.06±0.28
171	-	-	-	6.91±0.44	-	-	-	-	-	-	6.49±0.35
172	-	-	-	>10	-	-	-	-	-	-	7.33±0.42
173	-	-	-	5.30±0.25	-	-	-	-	-	-	5.66±0.34
174	-	-	-	5.11±0.41	-	-	-	-	-	-	6.16±0.37
175	-	-	-	5.62±0.35	-	-	-	-	-	-	6.01±0.42
176	-	-	-	>10	-	-	-	-	-	-	7.62±0.36
177	-	-	-	8.89±0.42	-	-	-	-	-	-	7.03±0.37
179	-	0.35±0.26	-	-	-	-	-	-	-	-	-
180	-	5.8±5.0	-	-	-	-	-	-	-	-	-
181	-	16±2	-	-	-	-	-	-	-	-	-
182	-	7.2±0.1	-	-	-	-	-	-	-	-	-
183	-	0.22±0.09	-	-	-	-	-	-	-	-	-
184	-	0.013± 0.004	-	-	-	-	-	-	-	-	-
185	-	0.0039 ± 0.0033	-	-	-	-	-	-	-	-	-
186	-	4.4±0.2	-	-	-	-	-	-	-	-	-
187	-	0.017±	-	-	-	-	-	-	-	-	-

		0.001									
188	0.5	0.4	-	0.6	-	-	-	-	-	0.65±0.16	-
189	0.25	0.2	-	0.25	-	-	-	-	-	0.25±0.03	-
190	3.29±0.74	-	-	-	-	-	-	-	-	-	-
191	3.13±0.03	-	-	-	-	-	-	-	-	-	-
192	16.52± 0.96	-	-	-	-	-	-	-	-	-	-
193	>20	-	-	-	-	-	-	-	-	-	-
194	9.06±0.04	-	-	-	-	-	-	-	-	-	-
195	8.99±0.65	-	-	-	-	-	-	-	-	-	-
196	9.48±0.07	-	-	-	-	-	-	-	-	-	-
197	8.21±0.67	-	-	-	-	-	-	-	-	-	-
198	12.08± 0.29	-	-	-	-	-	-	-	-	-	-
199	>20	-	-	-	-	-	-	-	-	-	-
200	19.09± 0.63	-	-	-	-	-	-	-	-	-	-
201	5.36±0.02	-	-	-	-	-	-	-	-	-	-
202	7.42±0.44	-	-	-	-	-	-	-	-	-	-
203	5.32±0.01	-	-	-	-	-	-	-	-	-	-
204	>20	-	-	-	-	-	-	-	-	-	-
205	9.12±0.30	-	-	-	-	-	-	-	-	-	-
206	18.68± 0.13	-	-	-	-	-	-	-	-	-	-
207	>20	-	-	-	-	-	-	-	-	-	-
208	>20	-	-	-	-	-	-	-	-	-	-
209	>20	-	-	-	-	-	-	-	-	-	-
210	19.08± 0.85	-	-	-	-	-	-	-	-	-	-
211	>20	-	-	-	-	-	-	-	-	-	-
212	>20	-	-	-	-	-	-	-	-	-	-

214	3.70±0.11	-	-	-	-	-	-	-	-	-	-
215	12.49± 0.10	11.70± 0.01	-	8.42±0.21	-	-	-	-	-	-	-
216	17.68± 0.27	14.82± 0.08	-	9.68±0.05	-	-	-	-	-	-	-
217	8.10±0.17	8.91±0.07	-	5.22±0.19	-	-	-	-	-	-	-
218	11.36± 0.22	11.59± 0.13	-	8.32±0.03	-	-	-	-	-	-	-
219	13.66± 0.32	13.9±0.12	-	8.91±0.04	-	-	-	-	-	-	-
220	8.67±0.13	8.56±0.05	-	7.54±0.05	-	-	-	-	-	-	-
221	6.61±0.13	5.29±0.04	-	4.58±0.43	-	-	-	-	-	-	-
222	9.66±0.05	11.11± 0.42	-	8.14±0.15	-	-	-	-	-	-	-
223	8.17±0.03	9.13±0.17	-	6.71±0.15	-	-	-	-	-	-	-
224	10.33± 0.09	17.17±0.0 3	-	13.20± 0.07	-	-	-	-	-	-	-
225	7.27±0.32	10.41± 0.09	-	4.82±0.13	-	-	-	-	-	-	-
226	5.84±0.18	4.53±0.07	-	2.36±0.32	-	-	-	-	-	-	-
227	21.21± 0.07	14.82± 0.08	-	15.47± 0.18	-	-	-	-	-	-	-
228	20.66± 0.01	17.39± 0.21	-	12.55± 0.11	-	-	-	-	-	-	-
229	16.71± 0.44	14.57± 0.23	-	9.18±0.25	-	-	-	-	-	-	-
230	6.81±0.14	17.37± 0.01	-	11.58± 0.26	-	-	-	-	-	-	-
231	9.51±0.18	16.17± 0.23	-	9.48±0.02	-	-	-	-	-	-	-
232	12.51± 0.47	14.17± 0.11	-	10.48± 0.22	-	-	-	-	-	-	-
233	-	-	-	-	-	-	-	-	-	-	0.9±0.1

Abbreviations:

A549= non-small-cell-lung cancer cell line

HeLa= human epithelial cervical cancer cell line

Bel-7402= human liver carcinoma cell line

MCF-7= human breast carcinoma cell line

HCT-8= human ileocecum carcinoma cell lines

H460-MX20=human non-small cell lung cancer (NSCLC) cell lines

BCRP/ABCG2= Breast cancer resistance protein / ATP-binding cassette subfamily G member 2

MV-4-11= leukemia cell lines dependent on FLT3-ITD

SK-OV-3=Human Ovarian Cancer Cell Line

KB-C2= an isogenic MDR1-overexpressing drug resistant cell line

MDR1= Multidrug Resistance Protein 1

HepG2= liver cancer cells

The most active 2 compounds for ABCG2 mechanism are written in purple (Compounds 41 and 80).

The most active 2 compounds for tubulin mechanism are written in red (Compounds 8 and 185).
